# Surface Chemical Compositions and Dispersity of Starch Nanocrystals Formed by Sulfuric and Hydrochloric Acid Hydrolysis

**DOI:** 10.1371/journal.pone.0086024

**Published:** 2014-02-27

**Authors:** Benxi Wei, Xueming Xu, Zhengyu Jin, Yaoqi Tian

**Affiliations:** 1 The State Key Laboratory of Food Science and Technology, School of Food Science and Technology, Jiangnan University, Wuxi, China; 2 Synergetic Innovation Center of Food Safety and Nutrition, Jiangnan University, Wuxi, China; RMIT University, Australia

## Abstract

Surface chemical compositions of starch nanocrystals (SNC) prepared using sulfuric acid (H_2_SO_4_) and hydrochloric acid (HCl) hydrolysis were analyzed by X-ray photoelectron spectroscopy (XPS) and FT-IR. The results showed that carboxyl groups and sulfate esters were presented in SNC after hydrolysis with H_2_SO_4_, while no sulfate esters were detected in SNC during HCl-hydrolysis. TEM results showed that, compared to H_2_SO_4_-hydrolyzed sample, a wider size distribution of SNC prepared by HCl-hydrolysis were observed. Zeta-potentials were −23.1 and −5.02 mV for H_2_SO_4_- and HCl-hydrolyzed SNC suspensions at pH 6.5, respectively. Nevertheless, the zeta-potential values decreased to −32.3 and −10.2 mV as the dispersion pH was adjusted to 10.6. After placed 48 h at pH 10.6, zeta-potential increased to −24.1 mV for H_2_SO_4_-hydrolyzed SNC, while no change was detected for HCl-hydrolyzed one. The higher zeta-potential and relative small particle distribution of SNC caused more stable suspensions compared to HCl-hydrolyzed sample.

## Introduction

Recently, there has been growing interest in starch nanocrystals (SNC) for abundant availability of starch, comparatively easy processability, biocompatibility, biodegradability, and non-toxicity, comparing with inorganic nanoparticles [1]. These properties make them as excellent candidates for implant materials and drug carriers. SNC have been also used as good reinforcing fillers in natural rubber, pullulan, polyurethane, polyvinyl alcohol, and soybean protein [2]. Furthermore, considering that the size of SNC is generally <100 nm (at least in one dimension) and SNC is readily surface-modified to obtain different hydrophobility [3], [4], SNC and their derivatives are deemed to be used as high-efficiency nanoparticle emulsifiers to prepare Pickering emulsions [5].

SNC are crystalline platelets resulting from the disruption of the semi-crystalline structure of starch granules by hydrolysis of amorphous parts. The preparation methods by different acid hydrolysis have been extensively studied [6]–[8]. At present, SNC are obtained generally by treating starch slurry with dilute sulfuric acid (H_2_SO_4_) or hydrochloric acid (HCl) at 25–55°C for various periods of time. However, hydrolysis of starch by H_2_SO_4_ was more widely adapted because of its high efficiency comparing with HCl [9], [10]. Furthermore, it was reported that SNC derived from H_2_SO_4_ hydrolysis resulted in more stable suspensions than HCl hydrolyzed samples [11]. This better stability was attributed to the surface chemical compositions of SNC, which reflected the interaction behaviors among SNC in aqueous or organic solvents.

A homogeneous dispersion state of SNC was a key step required for high mechanical performances of rubber or other material properties [12]. The electrostatic repulsive forces could also significantly affect the properties of Pickering emulsions [13]. Therefore, the surface chemical compositions of SNC played an important role in the application of different fields. So far, the change of surface chemical compositions of SNC after acid hydrolysis and the mechanism for better stability of SNC suspensions derived from H_2_SO_4_ have not been explicitly reported except for some hypotheses. These proposals mainly included the formation of sulfate ester groups on the surface of the SNC [9], the surface compositions of starch (such as lipids, protein and other metal ions carried by starch) making starch suspensions charge-stabilized [14], acquisition of more stable starch suspension after treating with high pressure homogenization due to higher zeta-potential of the colloids [15], and a stable and uniform SNC aqueous suspension prepared by introducing negative charges using crosslinking modification by sodium hexametaphosphate [16]. It could be concluded that the repulsive forces (expressed as zeta-potential) caused by surface chemical groups affected the dispersity of SNC.

In this work, the change of surface chemical compositions of SNC after H_2_SO_4_- and HCl- hydrolysis was, thus, analyzed and its effects on zeta-potentials of SNC suspensions were examined. This will provide basic information of SNC hydrolyzed with HCl and H_2_SO_4_ and explain the reason of their different dispersity.

## Materials and Methods

### Materials and Reagents

Waxy maize starch (WMS) was kindly donated by Tianjin Tingfung Starch Development Co., Ltd. (Tianjin, CHN). H_2_SO_4_, HCl, sodium hydroxide (NaOH), potassium chloride (KCl) and uranyl acetate were purchased from Sinopharm Chemical Reagent Co., Ltd. (Suzhou, CHN) and of analytical grade. Potassium bromide (KBr) was of spectroscopic grade and bought from Sinopharm Chemical Reagent Co., Ltd. (Suzhou, CHN). Milli-q water was used in all experiments.

### Preparation of Starch Nanocrystals

SNC were prepared by acid hydrolysis of waxy maize starch according to the method by Angellier et al. with minor modification [9]. Starch powders (50 g) were mixed with 500 mL of 3.16 M H_2_SO_4_ solution and placed at 40°C for 3 and 7 d under stirring at the speed of 200 rpm. For the HCl-hydrolyzed SNC, 20 g starch powders were mixed with 400 mL 2.2 N hydrochloric acid solution at 40°C for 7 d with constantly stirring at the speed of 200 rpm. The suspensions were washed by successive centrifugations in distilled water until pH was constant. The resultant suspensions were redispersed using Ultra Turrax T20 (IKA) at 13,500 rpm for 3 min to avoid aggregates and stored at 4°C with several drops of chloroform. The SNC concentrations were determined by weighting freeze-dried powders of the homogeneous dispersion of SNC (10 mL) and expressed as weight percentage relative to the volume of the water phase. The concentrations of H_2_SO_4_- and HCl-hydrolyzed SNC suspensions were 3.50% (w/v) and 2.08% (w/v), respectively. Different concentrations of SNC suspensions were derived by diluting the “stock” homogeneous dispersion of SNC.

### X-ray Photoelectron Spectroscopy (XPS) Analysis of SNC

XPS experiments were carried out by means of a Multifunctional imaging electron spectrometer (Thermo ESCALAB 250XI, USA). The spectrometer was equipped with a monochromatic Al Kα (*hν* = 1486.6 eV) X-ray source of 150 W at 15 kV. The kinetic energy of photoelectrons was determined with a hemispheric analyzer set to pass energy of 160 eV for wide-scan spectra and 20 eV for high-resolution spectra, respectively. During all measurements, electrostatic charging of the samples was avoided by means of a low-energy electron source working in combination with a magnetic immersion lens. Later, all recorded peaks were shifted by the same value to set the C1s peak to 284.8 eV. Quantitative elemental compositions were determined from peak areas using sensitivity factors experimentally and the spectrometer transmission function. Spectrum background was subtracted according to Shirley [17]. The high-resolved spectra were deconvoluted by means of a computer routine. Peaks were fitted to Gaussian-Lorentzian ratio (8∶2) curves after subtraction of baseline (Shirley method). SNC used for XPS analysis were lyophilized powders.

### Fourier Transform Infrared Spectroscopy (FT-IR) of SNC

The FT-IR spectra of the lyophilized and HCl- and H_2_SO_4_-hydrolyzed (for 3 d and 7 d) SNC powders were collected between 4000 cm^−1^ and 500 cm^−1^ on a FT-IR spectrophotometer (5DXC FT-IR, Nicolet Co. US) with 256 scans at a resolution of 4 cm^−1^. The SNC powders (3 mg) were ground with spectroscopic grade KBr (200 mg) powders and then pressed into 1 mm pellets. A blank disc was used as the background.

### Measurement of Zeta-Potentials of HCl- and H_2_SO_4_-hydrolyzed SNC Suspension

Zeta-potentials were determined in presence of 1 mM KCl at 25°C using a commercial Malvern Zetasizer Nano ZS 90. The pH of the suspension (0.01%, w/v) was adjusted using dilute HCl or NaOH. The SNC suspensions were placed for 2 h and 48 h before measurement. Measurements were carried out in quintuplicate for error analysis. The refractive index of fluid and particle used were 1.33 and 1.53, respectively.

### Transmission Electron Microscope (TEM)

TEM observations were performed using a JEOL JEM-2100 (HR) at an acceleration voltage of 80 kV. SNC suspensions (0.01%, w/v, pH = 6.5) hydrolyzed by HCl and H_2_SO_4_ were sonicated at 4°C for 15 min. A drop of suspension was spread onto the copper grids coated with carbon support film. Prior to complete drying, a drop of 2% (w/v) uranyl acetate negative stain was added. After 1 min, the liquid in excess was blotted with filter paper and the remaining film was allowed to dry for observation.

### Statistical Analysis

Statistical analysis was performed using ORIGIN 7.5 (OriginLab Inc., USA). Data were expressed as means ± standard deviations and analyzed by a one-way analysis of variance (ANOVA). P value ≤0.05 was regarded as significant throughout the study.

## Results and Discussion

### Surface Chemical Compositions of SNC

**Figure 1:**
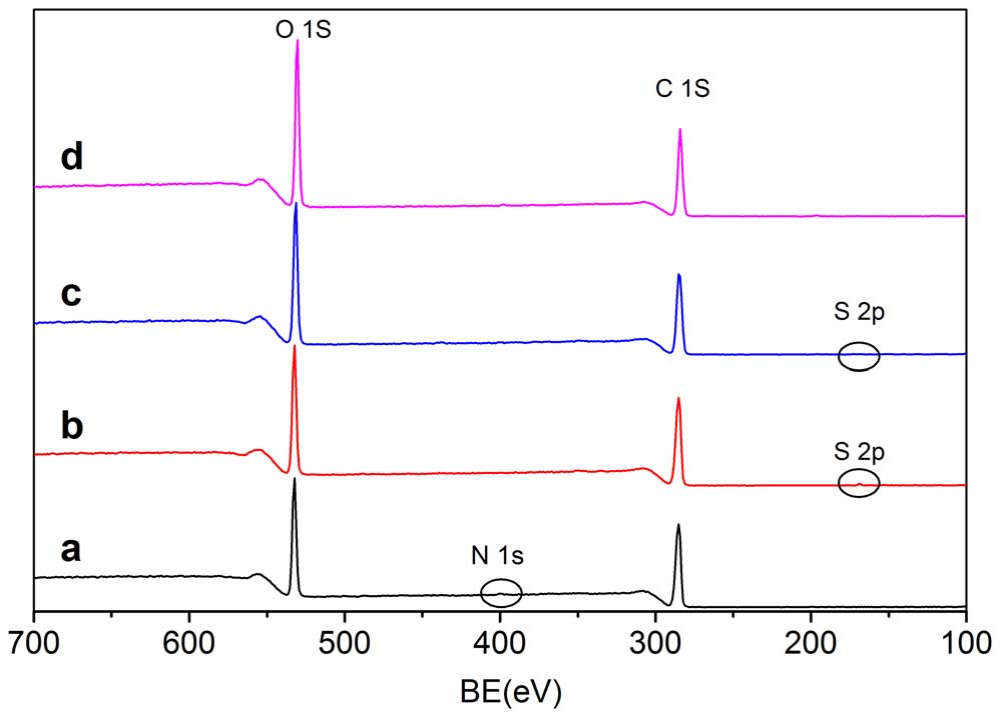
General XPS spectra of (a) WMS, (b) SNC_H2SO4,3_, (c) SNC_H2SO4,7_, and (d) SNC_HCl,7_.

The general XPS spectra of WMS and H_2_SO_4_-hydrolyzed starch for 3 d (SNC_H2SO4,3_) and 7 d (SNC_H2SO4,7_) were displayed in Fig. 1. The peaks around 531, 400, 285, and 168 eV were consisted with oxygen, nitrogen, carbon, and sulfur atoms, respectively. The elemental surface composition (%) and the oxygen-to-carbon ratios of the different samples were summarized in Table 1. For WMS, the oxygen/carbon (O/C) ratio was 0.43, which was lower than the theoretical O/C ratio (0.83). This indicated that the surface of WMS was composed of considerable amounts of other components except of polysaccharide. According to previous reports, the high carbon content was resulted from the surface lipids, because 0.08%–0.14% surface lipids (mainly free fatty acids) were presented in native waxy maize starch granules. Surface lipids were non-starch lipids in the cereal endosperm that strongly adsorbed to the surface layers of the starch granules during starch isolation [18], [19]. This demonstrated that surface lipids were the hydrocarbon impurities with strong adsorption behavior reported in previous studies [3], [20], [21]. Small amounts of nitrogen atoms were detected in the WMS due to the protein presented on the surface, which was in agreement with the report of Rindlav-Westling & Gatenholm [22]. With the hydrolysis process extending, the O/C ratio of SNC_H2SO4,3_ did not show significant change in the first 3 d, whereas it increased from 0.43 to 0.61 after hydrolysis for 7 d. The relative slow increase of O/C ratio in the first 3 days might result from the incomplete exposed internal reducing ends, since waxy maize starch could not absolutely crack. As the hydrolysis process extending, more and more internal reducing ends were exposed, leading to the increase of the O/C ratio. However, no significant changes of O/C ratio were detected between H_2_SO_4_- and HCl-prepared samples after hydrolyzing for 7 d.

**Table 1:** Elemental surface composition (atomic %) and oxygen to carbon ratio of starch, HCl- and H_2_SO_4_-hydrolyzed SNC.

Samples	O	C	S	N	O/C
WMS	30.08	69.30	-	0.52	0.43
SNC_H2SO4,3_	31.04	68.82	0.14	-	0.45
SNC_H2SO4,7_	37.70	62.23	0.07	-	0.61
SNC_HCl,7_	38.66	61.34	-	-	0.63

XPS spectra of SNC_H2SO4,7_ showed that the peak intensity corresponding to oxygen atoms became almost two times higher than the one corresponding to carbon atoms. This increase indicated the disruption of starch granules and the increase of reducing ends. The surface lipids that washed away by acid hydrolysis were contributed to the relative increase of oxygen atoms [3]. Small amounts of sulfur atoms were introduced to the surface of SNC after hydrolysis with H_2_SO_4_. No surface nitrogen was observed after acid hydrolysis, this result was in agreement with the findings reported by Thielemans, et al [23].

### Deconvolution of C 1 s and S 2p Peaks

**Figure 2:**
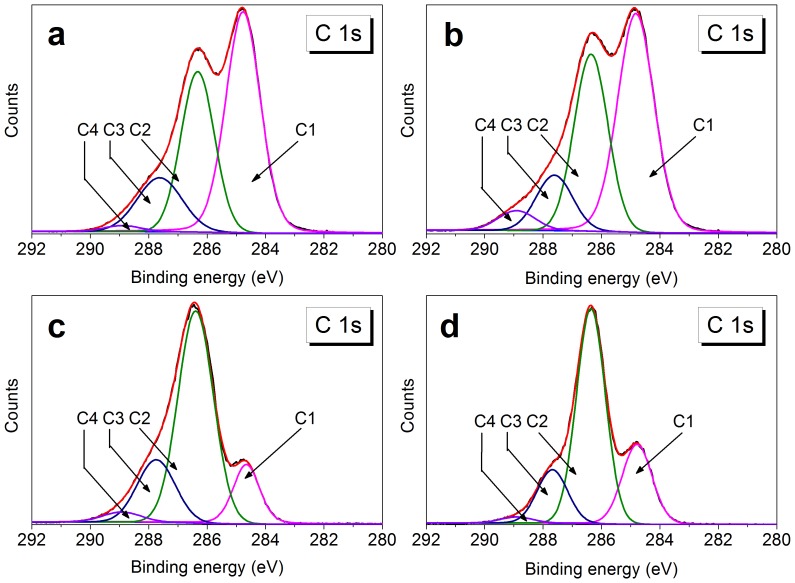
The high-resolution C 1 s deconvoluted XPS spectra of (a) WMS, (b) SNC_H2SO4,3_, (c) SNC_H2SO4,7_, (d) SNC_HCl,7_.

The C 1 s peak in the XPS spectra was deconvoluted for each sample into several peaks (Fig. 2). The different binding energies and relative percentages of the peaks were summarized in Table 2. For WMS, the C 1 s signal revealed four peaks at 284.8, 286.3, 287.6, and 288.9 eV arising from C1 (carbon atoms bonded only to carbon and/or hydrogen atom (C-C/C-H)), C2 (carbon atoms bonded to a single oxygen atom (C-O)), C3 (carbon atoms bonded to two noncarbonyl oxygen atoms or to a single carbonyl oxygen atom (O-C-O/C = O)), and C4 (carbon atoms bonded with a single oxygen atom and with a carbonyl oxygen (O-C = O)), respectively. The presence of C-C/C-H and O-C = O bonds further confirmed the lipids on the surface of WMS. After hydrolysis for 3 d, the relative content of C4 peak increased from 1.32% for WMS to 4.27% for SNC_H2SO4,3_, and the relative content of C1 peak decreased simultaneously. The increase in C4 peak might be ascribed to the adsorption of formic and levulinic acids derived from the hydrolyzed products of glucose [24], [25]. Formic acid had higher adsorption capacity and stronger affinity than levulinic acid [26]. Therefore, the increase of C4 peak (O-C = O) might mainly result from the adsorption of formic acid. The relative content of C4 peak decreased to 3.18% with the hydrolyzed time extending to 7 d. This may be due to an equilibrium adsorption state of organic acids and the complete removal of surface lipids during the hydrolysis process. These results were accorded with the previous report, in which carboxyl groups were detected in H_2_SO_4_-hydrolyzed cellulose microcrystal suspensions [27]. The relative content of C4 peak for SNC_HCl,7_ was lower than that for SNC_H2SO4,7_, the reason is probably that the concentration of H_2_SO_4_ used in the experiment was much higher than that of HCl.

**Table 2:** Surface functional group composition as obtained from the deconvolution of the C 1

Samples	C1	C2	C3	C4
	C-C/C-H	C-O	O-C-O/C = O	O-C = O
BE (eV)^a^	284.8	286.3±0.1	287.6±0.1	288.9±0.05
WMS	50.42	33.29	14.97	1.32
SNC_H2SO4,3_	49.23	34.94	11.56	4.27
SNC_H2SO4,7_	14.57	62.54	19.71	3.18
SNC_HCl,7_	23.60	59.10	15.49	1.83

a The binding energy for each signal is given with variation seen between different sample.

**Figure 3:**
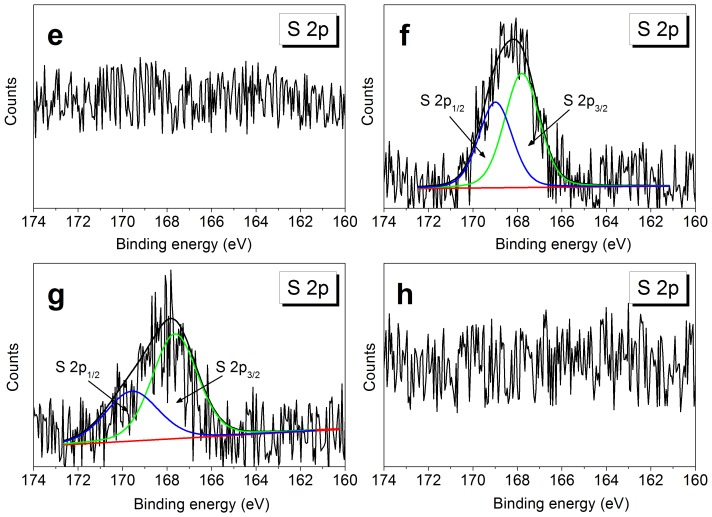
The high-resolution S 2p deconvoluted XPS spectra of (e) WMS, (f) SNC_H2SO4,3_, (g) SNC_H2SO4,7_, and (h) SNC_HCl,7_.

The high resolution S 2p peaks of WMS, SNC_H2SO4,3_, SNC_H2SO4,7_, and SNC_HCl,7_ were shown in Fig. 3. No surface sulfur was detected in WMS and SNC_HCl,7_. However, it was detected for SNC_H2SO4,3_ and SNC_H2SO4,7_ samples. The S 2p spectra at 168 eV were composed of S 2p3/2 and S 2p1/2 peaks, and both peaks represented sulfur in its highest oxidation state, i.e., O-SO_3_^−^ or C-SO_3_^−^. Nevertheless, no C-S bond was presented in the C 1 s peaks for SNC_H2SO4,3_ and SNC_H2SO4,7_ (Figs. 2b/c), indicating sulfate esterification of hydroxyl bonds and H_2_SO_4_ (Figs. 3f/g). The formation of sulfate esters was previously reported for cellulose nanocrystals, while the details were not shown for SNC except for some hypothesis [9], [28]. The relative atomic percentages of sulfur reduced from 0.14% for SNC_H2SO4,3_ to 0.07% for SNC_H2SO4,7_. This decrease indicated that sulfate esters were probably hydrolyzed with the hydrolyzed process extending.

### FT-IR Analysis of SNC

**Figure 4:**
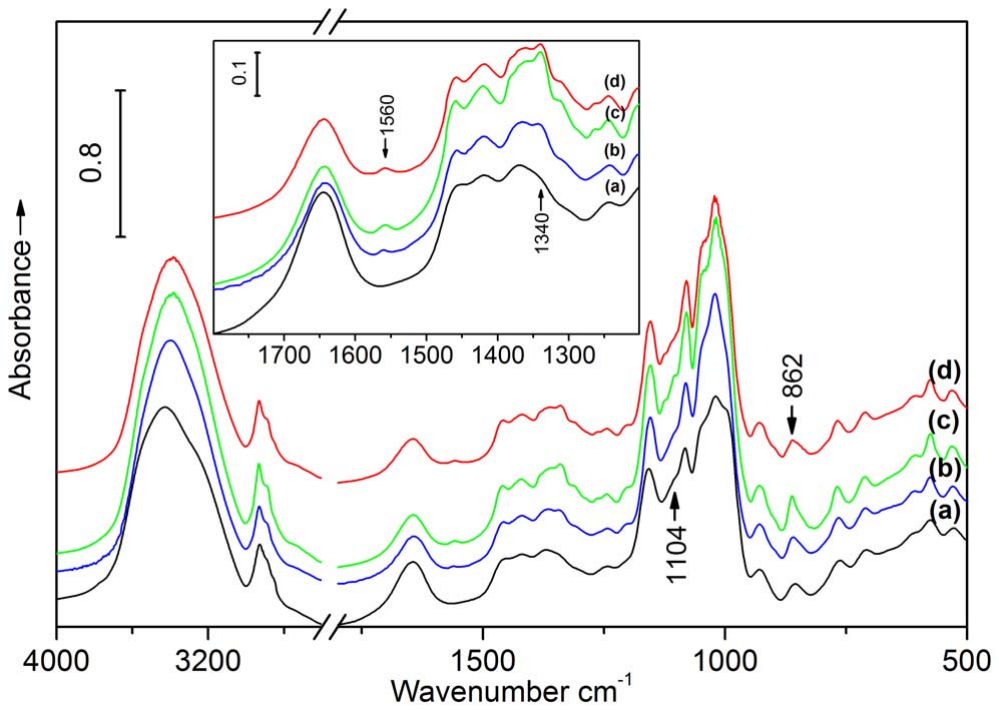
FT-IR spectra of (a) WMS, (b) SNC_H2SO4,3_, (c) SNC_H2SO4,7_, and (d) SNC_HCl,7_.

Fig. 4 illustrated FT-IR spectra of waxy maize starch and HCl- and H_2_SO_4_-hydrolyzed SNC. For WMS (Fig. 4a), a typical spectrum enclosed absorption bands at 3435 cm^−1^, revealing the O-H stretching vibration of -OH groups in the glucose units, and the peaks at 2929 cm^−1^, 1450 cm^−1^ and 1370 cm^−1^ ascribe to the C-H stretching and bending modes of the methylene. The peak at 1648 cm^−1^ originated from the bending vibration of H-O-H in the absorbed H_2_O. The peaks at 1155 cm^−1^, 1080 cm^−1^, and 1020 cm^−1^ reflected the stretching mode of C-O-C linkages in the glucosidic rings [29]. For the HCl- and H_2_SO_4_-hydrolyzed SNC, the emergence of two new bands adsorbing at 1560 cm^−1^ and 1340 cm^−1^ could be ascribed to the asymmetric and symmetric stretching of -COOH. The adsorbing bands shifted to lower wavenumbers compared to previous reports [30]. This difference was probably because of hydrocarbon chain effect from levulinic acid lowed the adsorbing band of carboxylate groups [31], and the resulted adsorbing peaks at 1560 and 1340 cm^−1^ were overlapped of formic and levulinic acids. As the hydrogen bonds were formed between carboxylate groups and SNC, which reduced the adsorbing bands of carboxylate groups [32]. Therefore, the present band at 1644 cm^−1^ might be a combination of carbonyl groups and absorbed H_2_O [33]. It was obvious that the band at 1644 cm^−1^ for WMS was considerably narrower than HCl- and H_2_SO_4_-hydrolyzed samples, which confirmed the interaction of carbonyl groups and -OH through hydrogen bonds. These results suggested that formic and levulinic acids were adsorbed on the surface of SNC.

In addition, the band at 862 cm^−1^ for SNC_H2SO4,7_ was stronger than that of the other samples, indicated that an overlapping band existed in the region of 823 cm^−1^–881 cm^−1^ (Fig. 4c). The overlapping band could be assigned to the vibration of C-O-S bond, which was in a good agreement with values reported in the literature [34]. Otherwise, the slight increase of the adsorbing band at 1104 cm^−1^ attributed to sulfate also proved possibility of the formation of sulfate ester.

### Reactions Occurred During Acid Hydrolysis

**Figure 5:**
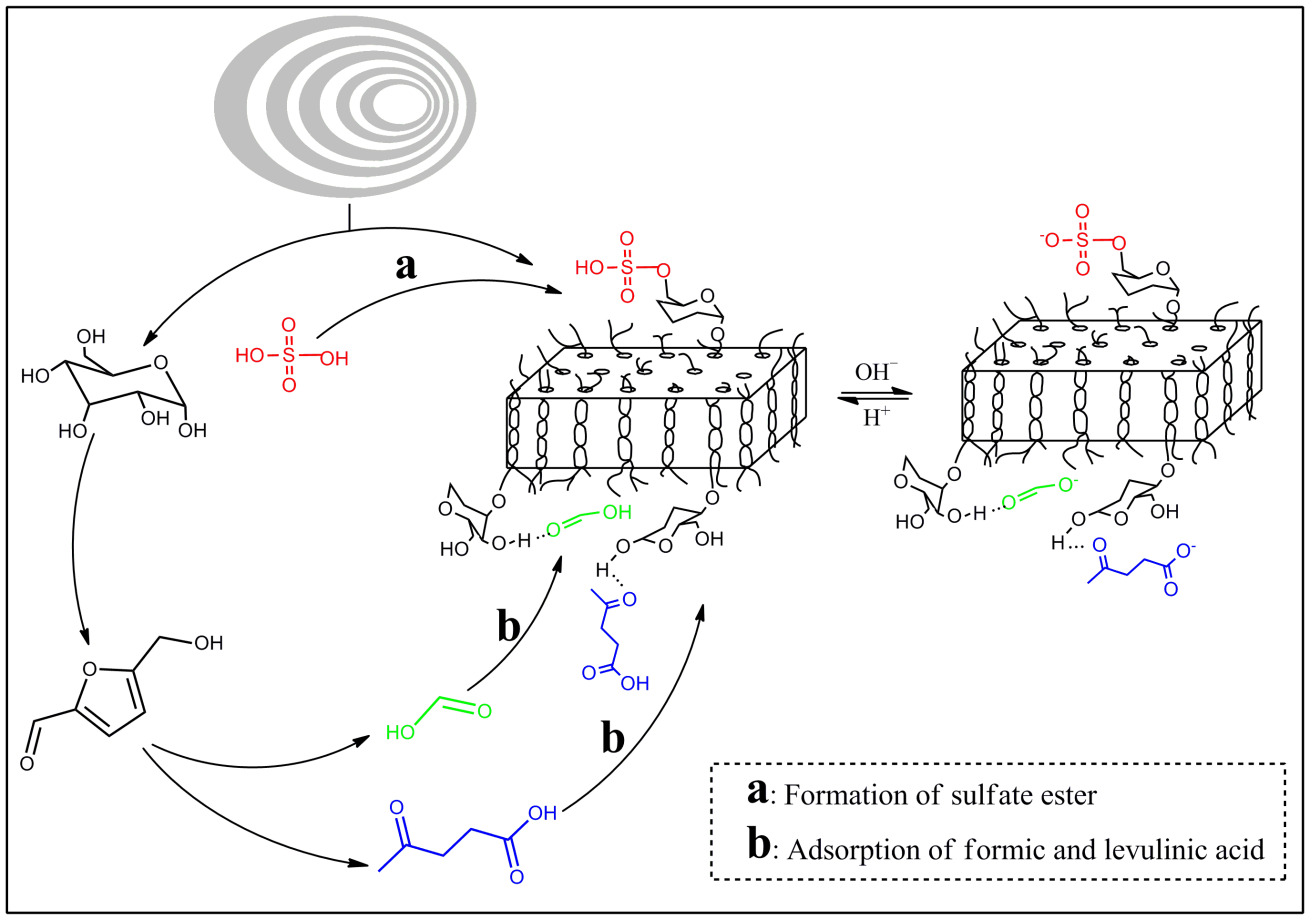
Formation of sulfate esters and adsorption of formic and levulinic acids on the surface of SNC (a: Formation of sulfate ester, b: adsorption of formic and levulinic acids).

According to the above analysis, it could be concluded that three major reactions occurred during acid hydrolyzed process. (1) Breakage of α-D-(1-4) and α-D-(1-6)-glycosidic bonds was the dominant reaction, which resulted in disappearance of amorphous and semicrystalline layers of the WMS, leaving the acid-resistant fraction named SNC; (2) Glucose was further degraded to formic and levulinic acids, and adsorption of these acids to the surface of SNC made them negative-charged (Fig. 5); (3) Formation of sulfate ester groups on the surface of the SNC, and the esterification reaction occurred simultaneously with the hydrolysis of the sulfate ester.

### Zeta Potential of H_2_SO_4_- and HCl-hydrolyzed SNC Suspensions

**Figure 6:**
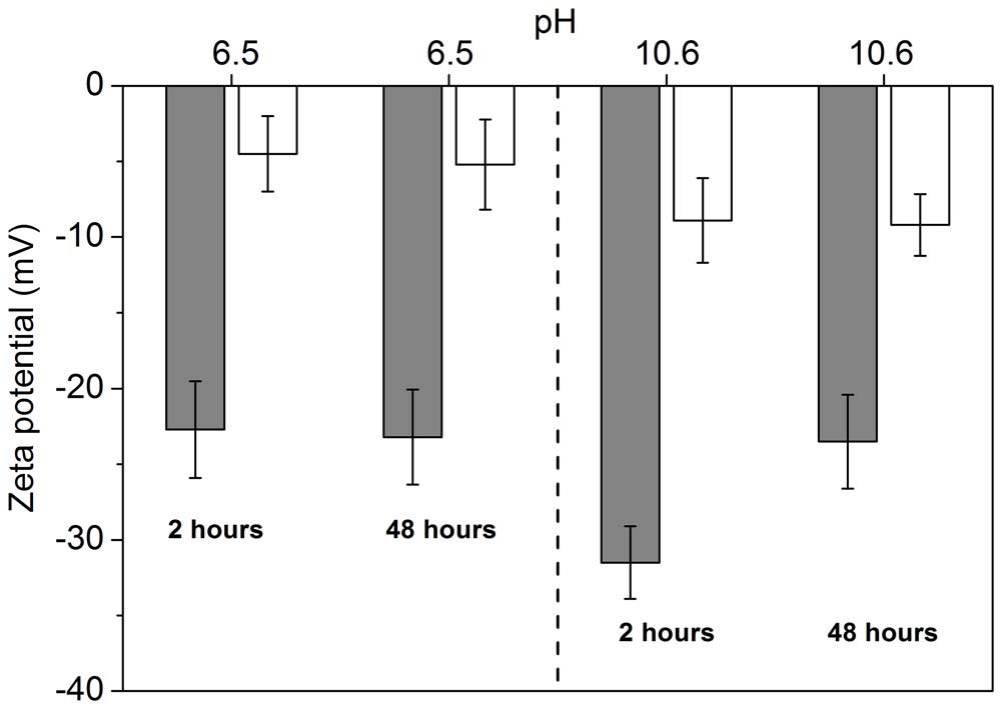
Zeta potential of SNC_H2SO4,7_ (gray color) and SNC_HCl,7_ (white color) suspensions at pH 6.5 and 10.6 placed for 2 h and 48 h, respectively. Data are represented as the mean ± standard deviation (n = 3).

Zeta-potentials of SNC_HCl,7_ and SNC_H2SO4,7_ suspensions at pH 6.5 and 10.6 placed for 2 and 48 h were shown in Fig. 6. For the SNC_HCl,7_ suspension, the final pH was 6.5 with complete washing by distilled water. However, it was difficult to wash SNC_H2SO4,7_ till neutrality due to presence of carboxyl groups and sulfate esters (Fig. 5). Therefore, the dispersion pH of SNC_H2SO4,7_ suspension was adjusted to 6.5 with dilute NaOH in this work. Owing to the presence of sulfate esters and carboxyl groups, zeta-potential of SNC_H2SO4,7_ reached −23.1 mV, which was much lower than that of the SNC_HCl,7_ suspension. No changes of zeta-potentials were detected for the two samples after 48 h at pH 6.5. Zeta-potentials decreased to −32.3 and −10.2 mV for SNC_H2SO4,7_ and SNC_HCl,7_, respectively, while the dispersion pH was adjusted to 10.6 and placed for 2 h. After 48 h, zeta-potential for SNC_H2SO4,7_ increased to −24.1 mV, while no changes were found for SNC_HCl,7_. These results revealed that degradation of sulfate esters under alkaline condition contributed to the increase of the zeta-potential of SNC_H2SO4,7_ suspension.

### TEM and Sedimentation Properties of HCl- and H_2_SO_4_-hydrolyzed SNC Suspensions

**Figure 7:**
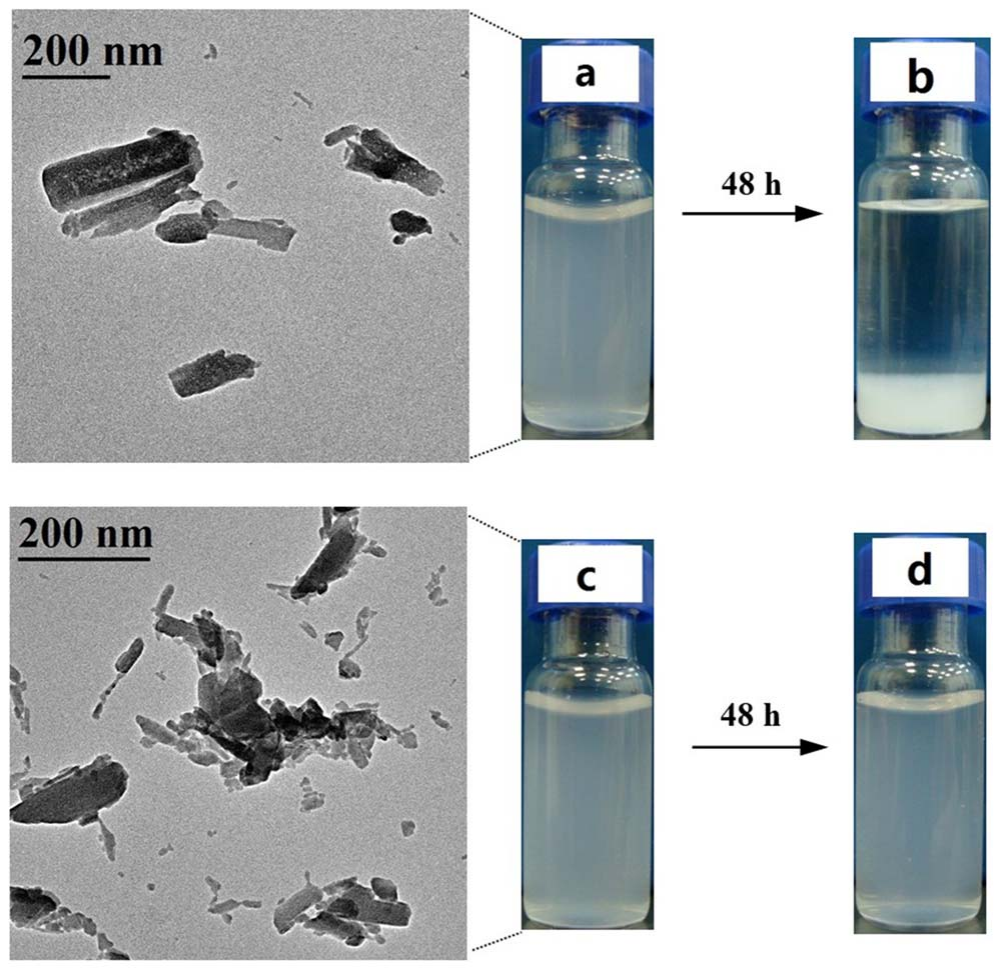
TEM pictures and sedimentation properties of HCl (a and b) and H_2_SO_4_ (c and d) hydrolyzed SNC suspensions after 2 h and 48 h.

Fig. 7 shows TEM pictures and sedimentation properties of HCl- and H_2_SO_4_-hydrolyzed SNC suspensions. It could be seen from the picture that SNC derived from HCl-hydrolysis for 7 d showed a wide particle distribution, which was in the range of 50–300 nm. The low acid concentration used in the experiment might result in the heterogeneous SNC distribution. On the contrary, a homogenous SNC distribution in the range of 50–150 nm was obtained after H_2_SO_4_-hydrolysis for 7 d.

In addition, the aggregation behavior of HCl-hydrolyzed SNC was more serious than H_2_SO_4_-hydrolyzed sample. This could be ascribed to the relatively low zeta-potential of SNC suspension obtained by HCl-hydrolysis. Compared to the SNC_HCl,7_ suspension, the higher zeta-potentials and the relative smaller particle distribution of SNC_H2SO4,7_ generated the more stable suspensions.

## Conclusions

Surface chemical compositions of H_2_SO_4_-hydrolyzed SNC were different from the HCl-hydrolyzed samples. Adsorption of formic and levulinic acids forming from the acid hydrolysis of glucose were probably contributed to the presence of carboxyl groups in HCl- and H_2_SO_4_-hydrolyzed SNC, while sulfate esters in H_2_SO_4_-hydrolyzed SNC were derived from the esterification reaction between hydroxyl bonds and H_2_SO_4_. The higher zeta potential for SNC_H2SO4,7_ and the samller particle distribution limited flocculation and generated more stable suspensions comparing with the HCl-hydrolyzed sample. These results suggested that a more stable suspension was obtained by H_2_SO_4_-hydrolysis due to the higher zeta potential and much smaller and narrower particle size distribution.
